# Investigation of possible molecular mechanisms underlying the regulation of adhesion in *Vibrio alginolyticus* with comparative transcriptome analysis

**DOI:** 10.1007/s10482-015-0411-9

**Published:** 2015-03-01

**Authors:** Wendi Kong, Lixing Huang, Yongquan Su, Yingxue Qin, Ying Ma, Xiaojin Xu, Mao Lin, Jiang Zheng, Qingpi Yan

**Affiliations:** 1 Key Laboratory of Healthy Mariculture for the East China Sea, Ministry of Agriculture, Fisheries College, Jimei University, Xiamen, 361021 Fujian People’s Republic of China; 2 College of Ocean & Earth Sciences, Xiamen University, Xiamen, 361005 Fujian People’s Republic of China

**Keywords:** *Vibrio alginolyticus*, Adhesion, Transcriptome

## Abstract

Adhesion capability to fish mucus, which can be affected by environmental conditions, is considered to be a key virulence factor of *Vibrio alginolyticus* although the molecular mechanism is still unclear. In the present study, *V. alginolyticus* was treated with stress conditions including Cu^2+^ (50 mg/L), Pb^2+^ (100 mg/L), Hg^2+^ (50 mg/L) and low pH (pH 5). We found these stress treatments were capable of reducing the adhesion of *V. alginolyticus*, while the expression levels of multiple genes were significantly changed according to the results of high throughput sequencing. The expression of randomly selected genes was confirmed by QPCR, which reinforced the reliability of the sequencing data. Ontology assignments and KEGG pathway analysis indicated that stress treatments affect pathways that may be related to adhesion. Our results identified genes which might play a key role in the adhesion process of *V. alginolyticus*, which could lay a foundation for further functional analysis of these genes in the process of adhesion. As these genes were sensitive to environmental factors, this may explain why the adhesion process can be influenced by environmental factors.

**Electronic supplementary material:**

The online version of this article (doi:10.1007/s10482-015-0411-9) contains supplementary material, which is available to authorized users.

## Introduction

*Vibrio alginolyticus*, an important opportunistic pathogen for marine organisms (Carli et al. 1993), is associated with epidemic vibriosis which causes mass mortality to many marine cultured animals, including fish (Lee et al. 1995; Balebona et al. 1998), shellfish (Liu et al. 2001), shrimp (Lee et al. 1996) and coral reefs (Xie et al. 2013). *V. alginolyticus* is also a pathogenic bacterium for people who are in contact with seafood, which may lead to otitis and wound infections (Lin et al. 2001). In recent years, *V. alginolyticus* has been frequently found to be the cause of disease in marine cultured fish in China and has resulted in considerable losses (Wang et al. 2001).

Bacterial adhesion to fish tissue surfaces is an important step in the initial stage of infection (Thune et al. 1993). Intestines and injured skin are considered to be the portals of entry for *Vibrio* species into fish (Chen et al. 2008). A mucous layer can be found covering the intestine and skin of fish. Bacterial adherence to the mucus is an essential requirement for infection by different pathogens (Speare et al. 1992). Thus, the ability to adhere to mucus is considered to be a crucial bacterial virulence mechanism (Chen et al. 2008). Bacterial adhesion is influenced by bacterial factors, the adhesion substrate and environmental factors (Yan et al. 2007). In our previous research, we detected effects of environmental factors (including Cu^2+^, Pb^2+^, Hg^2+^, low pH, high pH, low salinity, high salinity, low temperature, and high temperature) on *V. alginolyticus* adhesion. Our results showed that Cu^2+^, Pb^2+^, Hg^2+^ and low pH could reduce the adhesion of *V. alginolyticus.*

As an important environmental factor, pH has a great effect on bacterial attachment (Balebona et al. 1995; Yan et al. 2007). Additional research in our laboratory indicated that low pH could reduce the adhesion of *V. alginolyticus* to skin mucus of large yellow crokers (data not shown). Moreover, our research also indicated that heavy metals, including Cu^2+^, Pb^2+^, and Hg^2+^, could reduce the adhesion of *V. alginolyticus* to skin mucus of large yellow crokers at different concentrations (Fig. S1).

Although several genes have been shown to be associated with vibrial adhesion (Qin et al. 2013), the mechanism of bacterial adhesion is still unclear. Additionally, little is known about how the adhesion process can be influenced by environmental factors. A powerful approach to determine how an organism responds to a particular abiotic condition is to determine how it changes the expression of its genes (Simon et al. 2013). Traditional techniques are time consuming and impractical for large scale detection of hundreds of genes. Instead, high throughput sequencing has been widely used in bacterial transcriptome profiling, which can simultaneously determine expression levels for large numbers of genes in a single experiment and help to gain insight into molecular mechanisms in bacteria (Xu et al. 2003; Bisharat et al. 2013; Lenz et al. 2011; Sharma et al. 2010; Arnvig et al. 2011; Yang et al. 2009). Although the complete genome of *V. alginolyticus* has been sequenced, the transcriptome analysis of *V. alginolyticus* has not yet been reported.

In the present research, we present the first deep sequencing study of the transcriptome of *V. alginolyticus* cultured under normal and stress conditions such as Cu, Pb, Hg and low pH. The objectives of this study were to: (1) determine a broad spectrum of expression of genes associated with bacterial adhesion in order to offer new clues for further understanding of the mechanism(s) underlying the regulation of adhesion in *V. alginolyticus*; (2) to gain further understanding of how the adhesion process can be influenced by environmental factors.

## Materials and methods

### Bacterial samples and culture conditions

*Vibrio alginolyticus* (ND-01) was previously isolated from a naturally infected large yellow croaker by our laboratory and confirmed as pathogenic by artificial infection (Yan et al. 2001). The sample was stored at −80 °C in physiological saline with 10 % glycerol. Bacteria were cultured on tryptic soy agar (TSA) supplemented with 2 % NaCl at 28 °C. Bacteria were challenged by chemical stresses including Cu^2+^ (50 mg/L CuSO_4_·5H_2_O), Pb^2+^ (100 mg/L (CH3COO)_2_Pb), Hg^2+^ (50 mg/L HgCl_2_) and low pH (HCL was used to lower the pH to pH 5), respectively. These conditions were chosen based on our earlier research, which investigated the effects of these stresses on adhesion at different concentrations Supplementary Fig. 1). The control group was cultured on normal TSA slant (pH = 7). There were three replicates for each of the treatments.

### Mucus preparation

Healthy large yellow croakers were obtained from marine culture-cages, at Ningde in Fujian province of China. Intestinal mucus was prepared using a method modified from one described before (Chen et al. 2008). The intestines were removed and transferred to sterile petri dishes and washed with sterile PBS (0.01 mol/L pH 7.2). Then the guts were split open with a scalpel. The intestine mucus was harvested by scrapping off the inner surface of the intestines with a plastic spatula to remove the mucus gel layer covering the intestinal lumen and homogenized in PBS. The mucus preparations were centrifuged twice at 20,000 g, 4 °C for 30 min to remove particulate materials. The final supernatant was filtered through 0.45 and 0.22 μm pore size filters. The mucus samples were adjusted to 1 mg protein/mL PBS. The protein concentration was determined using the method of Bradford (Bradford et al. 1976).

### In vitro adhesion assay

The bacterial adhesion assay was conducted following the method described by Yan et al. (2006). Briefly, 50 μL of mucus suspension were evenly spread on a 22 × 22 mm area of glass slides and fixed by methanol for 20 min, after the mucus was dry. *V. alginolyticus* ND-01 was grown in TSB liquid medium supplemented with 2 % NaCl overnight. Then, the bacteria were collected by centrifugation and suspended in PBS. The suspensions were adjusted to 10^8^CFU/ml according to the values of OD_560_ (based on a linear relationship between OD_560_ values and the CFU values of the bacterial suspension). Then, 1 mL aliquots of bacterial suspensions (10^8^CFU/ml) was placed on mucus-coated glass slides, incubated at 25 °C for 2 h, and then washed thoroughly five times with PBS. Finally, slides were fixed with 4 % methanol for 30 min and stained with crystal violet for 3 min. After staining, the slide was observed using a microscope and imaged with a digital video camera (magnification, ×1000). The number of bacteria was quantified using IPwin software from the images (n = 3 slides per condition, 20 fields of view per slide).

### Total RNA extraction and cDNA library construction

Head-on comparison of RNA-Seq with microarrays has shown that RNA-Seq has negligible technical variability, making it possible to obtain a reliable estimate of gene expression without replicate analysis (Marioni et al. 2008; Reddy et al. 2012). Therefore, we applied RNA-Seq and conducted the analysis without a replicate. rRNA was removed with a Ribo-Zero rRNA Removal Kit after total RNA was collected from the pooled bacteria. mRNA was disrupted into fragments, which were used for the first-strand cDNA synthesis. The second-strand cDNA was synthesized using buffer, dATPs, dGTPs, dCTPs, dUTPs, RNase H and DNA polymerase I respectively. Short fragments were purified with a QiaQuick PCR extraction kit and resolved with EB buffer for end reparation and adding poly(A). After that, the short fragments were connected with sequencing adapters. Then, the UNG enzyme was used to degrade the second-strand cDNA, and the product was purified using a MiniElute PCR Purification Kit before PCR amplification.

### Illumina sequencing and data processing

The amplification products were sequenced using Illumina HiSeq2000. Dirty raw reads which contain adapters, unknown or low quality bases were discarded to obtain clean reads. Clean reads were mapped to the reference genome and gene sequences respectively using SOAP2 (Li et al. 2009). Mismatches (≤5 bases) were allowed in the alignment. The unigene expression was calculated in RPKM (Reads Per kb per Million reads) method (Mortazavi et al. 2008), which can eliminate the influence of different gene length and sequencing discrepancy on the calculation of gene expression and therefore the difference of gene expression among samples can be compared. Differential expression genes (DEGs) analysis was applied to identify differentially regulated genes (different ratio ≥2) between stressed samples and the control, using the two classes unpaired MA-plot-based method to detect and visualize gene expression difference with significant *P* value <0.001.

The data have been deposited in the NCBI Sequence Read Archive (SRA) and can be accessed through accession number SRP049226.

### Functional classification and enrichment analysis for DEGs

For DEGs annotation, we used the Blast2GO program to obtain GO annotation of the unigenes. After acquiring the GO annotation for every gene, we used WEGO software to carry out GO functional classification for all genes and understand the distribution of gene functions of the species at the macro level. The calculated *P* value went through Bonferroni Correction, taking corrected *P* value ≤0.05 as a threshold. GO terms fulfilling this condition were defined as significantly enriched GO terms in DEGs.

The COG and KEGG pathways annotation was carried out using Blastall software against COG (http://www.ncbi.nlm.nih.gov/COG) and KEGG (http://www.genome.jp/kegg/) database. Q value was defined to be the FDR analogue of the *P* value. Pathways with Q value ≤0.05 were regarded as significantly enriched in DEGs.

### QPCR assay

In order to further validate the results of sequencing, expression levels of genes were verified by QPCR (n = 3). QPCR analysis was performed on a Rotor-gene6000 Real-Time PCR system (ABI, USA) using SYBR green I fluorescent dye. The reactions were performed in a 10 µL volume mix containing 0.2 µL SYBR Green I, 5 pmol/L specific primers and approximately 50 ng cDNA. The cycling parameters were 95 °C for 10 min, followed by 45 cycles of 95 °C for 20 s, 55 °C for 20 s, and 72 °C for 20 s. Threshold cycles and dissociation curves were determined with Rotor-gene6000 software, to confirm that only one PCR product was amplified and detected, and gene expression levels were normalized to 16S RNA (which showed an invariant expression under the experimental conditions). Primer sequences designed using software Primer Premier 5.0 are listed in Table S1.

### Data processing

Results were reported as mean ± SD The data were statistically analyzed with one-way ANOVA followed by Dunnett’s multiple comparison tests via SPSS 13.0 software. A value of *P* < 0.05 was used to indicate significant difference.

The Relative Expression Software Tool (REST 2008-version 2) was used to calculate the relative expression of mRNA target genes in real time fluorescence quantitative PCR using the Pair Wise Fixed Reallocation Randomization Test (Pfaffl et al. 2002). The mathematical model used was based on the mean crossing point deviation between the sample and the control group, normalized by the mean crossing point deviation of the reference genes. Specific amplification efficiencies were included in the correction of the quantification ratio. Significant differences between groups were determined by ANOVA followed by the Tukey’s LSD.

## Results

### Adhesion of *V. alginolyticus* after stress

*V. alginolyticus* ND-01 exhibited variable adhesion to the intestinal mucus of large yellow crokers after stress challenges. The adhesion of the low pH treated bacteria significantly decreased to 43.4 %, while Cu^2+^, Pb^2+^, and Hg^2+^ treated groups significantly decreased to 62.6, 60.7 and 59.4 %, respectively (Table 1).

**Table 1:** The adhesion capacity to mucus of wild and stressed *V. alginolyticus*

	Control	Cu	Pb	Hg	Low pH
Cells/vision	420.0 ± 46.7	262.9 ± 29.2^*^	255.1 ± 28.4^*^	249.3 ± 27.7^*^	182.4 ± 20.3^*^

^*P < 0.05 versus the control group^

### Mapping of reads and identification of DEGs

To compare the transcriptome profile of stressed groups with the control, RNA sequencing libraries were constructed for Cu^2+^, Pb^2+^, Hg^2+^, low pH treated bacteria and the control. Each library generated about 12.9–13.8 million reads (Table 2), which were mapped to the *V. alginolyticus* E0666 genome sequence (NCBI Reference Sequence: NZ_AMPD00000000.1). For Cu^2+^, Pb^2+^, Hg^2+^, low pH treated groups and the control, about 87.5, 87.7, 88.4, 88.1 and 87.2 % reads were matched to NCBI annotated gene regions, respectively (GEO database: accession number GSE44215) (Table 2). The distribution of gene coverage among the different groups was also determined (Table S2). There were 4565 protein-coding genes predicted in the genome of *V. alginolyticus* E0666 by genome analysis. In total, 4045 transcripts were identified in this study. With a threshold of more than five reads mapped to the transcripts of a given gene in each sample, expression of a total of 3962, 3924, 3978, 3987 and 3994 protein-coding genes was detected in Cu^2+^, Pb^2+^, Hg^2+^, low pH treated groups and the control, respectively.

**Table 2:** Overview of reads distribution

Reads (million)	Control	Cu	Pb	Hg	Low pH
Total reads	13.3	12.9	13.1	13.8	13.4
Total mapped reads	11.6	11.3	11.5	12.1	11.4

The R-package DEG-seq was used to identify DEGs. The list of genes with significantly different expression levels was refined using the criterion of *P* value ≤0.001 in t tests. These analysis finally yield 1637 (177 up regulated and 1460 down regulated), 1085 (74 up and 1011 down), 846 (143 up and 703 down) and 1791 (253 up and 1538 down) DEGs in Cu^2+^, Pb^2+^, Hg^2+^, and low pH treated groups, respectively.

**Fig. 1:**
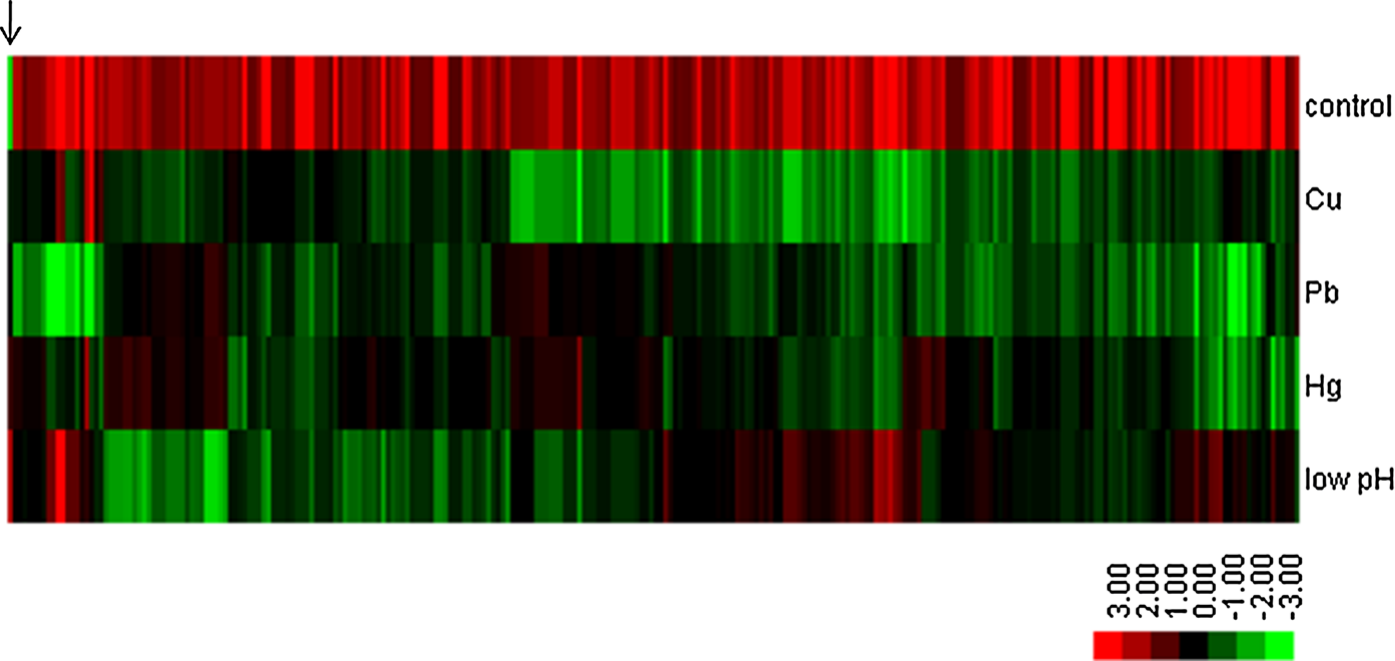
Hierarchical clustering of commonly changed DEGs. *Green* and *red* indicate decreased and increased expression, respectively. Transcripts were clustered by hierarchical clustering using the complete linkage algorithm and Pearson correlation metric in R. The *arrow* indicates the common downregulated gene

Only one up-regulated gene was found in all four treated groups, while 212 genes were down-regulated in all four treated groups (Table S3). These genes were hierarchically clustered and used to produce a heat map (Fig. 1). The heat map display the alteration trend of these 213 common regulated genes.

### Quantitative real-time PCR (QPCR)

**Fig. 2:**
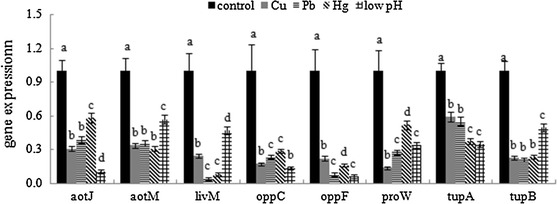
QPCR analysis of the expression of randomly selected novel genes. Data are presented as mean ± SD (n = 3). Means of treatments not sharing a common letter are significantly different at *P* < 0.05

In order to validate the results of sequencing, we performed QPCR on randomly selected DEGs. The results of QPCR matched those of the sequencing: all treatments significantly down-regulated the expression of *aotJ, aotM, livM, oppC, oppF, proW, tupA* and *tupB* (Fig. 2). These data reinforced the reliability of the sequencing data.

### Gene ontology (GO) assignments of the DEGs

**Fig. 3:**
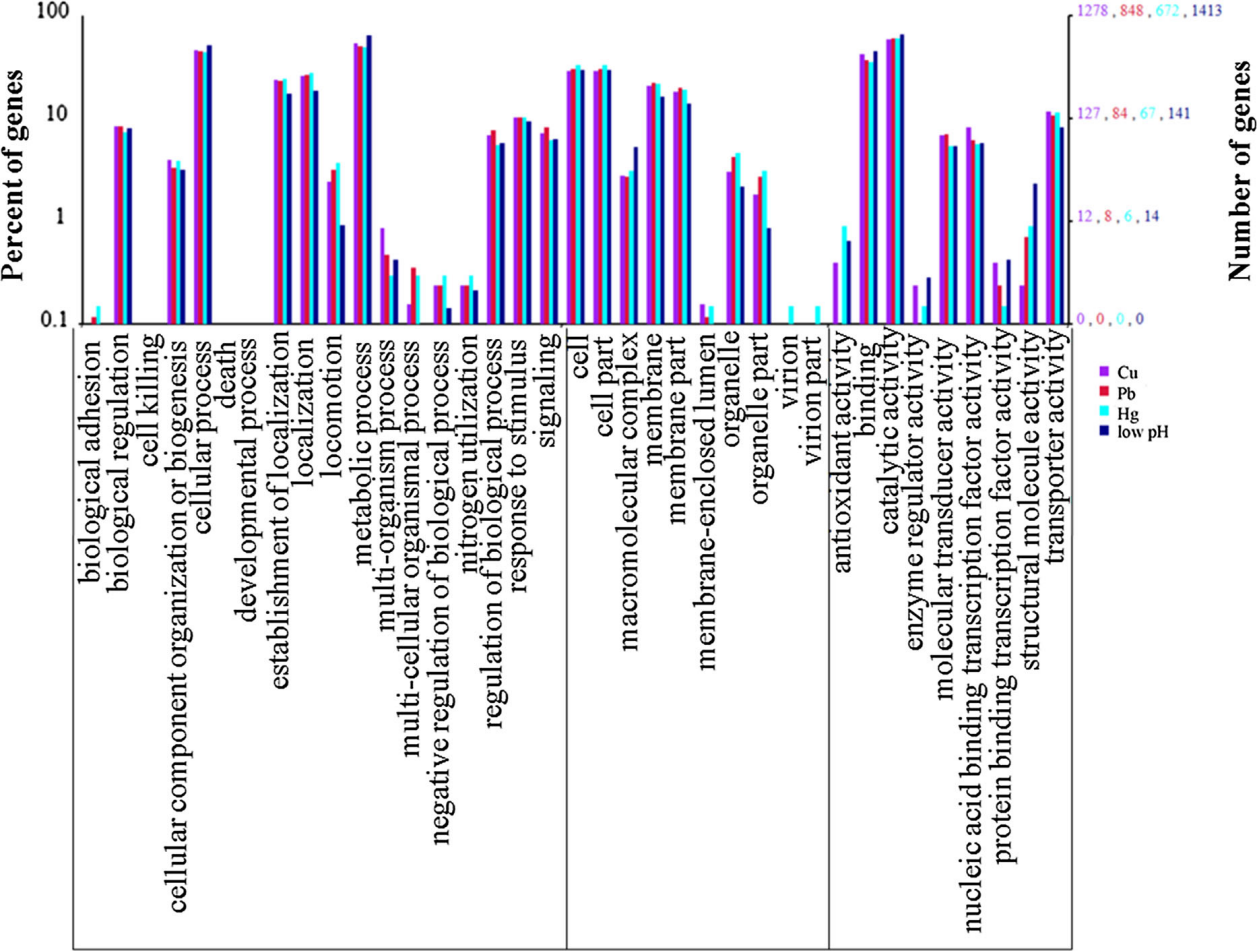
Functional annotation of DEGs based on known proteins in the database. Each annotated sequence was assigned at least one GO term. GO terms at the second level were displayed to classify the results based on their involvement in biological processes, molecular functions, and cellular components

The functions of DEGs were analyzed according to GO, while the number of genes mapped to every term was calculated. According to this analysis, 1278, 848, 672 and 1413 DEGs from Cu^2+^, Pb^2+^, Hg^2+^, and low pH treated groups were categorized into 37 enriched functional groups (Fig. 3).

Analysis of GO categories showed that the functional distribution of the DEGs from each stressed group was similar. In the libraries, most of the corresponding biological process genes were involved in cellular processes, metabolic processes, establishment of localization and localization. Most of the cellular component genes encoded proteins associated with cell, cell part, membrane and membrane part; and most of the molecular function genes were associated with binding and catalytic activity (Fig. 3).

### Clusters of orthologous groups (COG) of DEGs

**Fig. 4:**
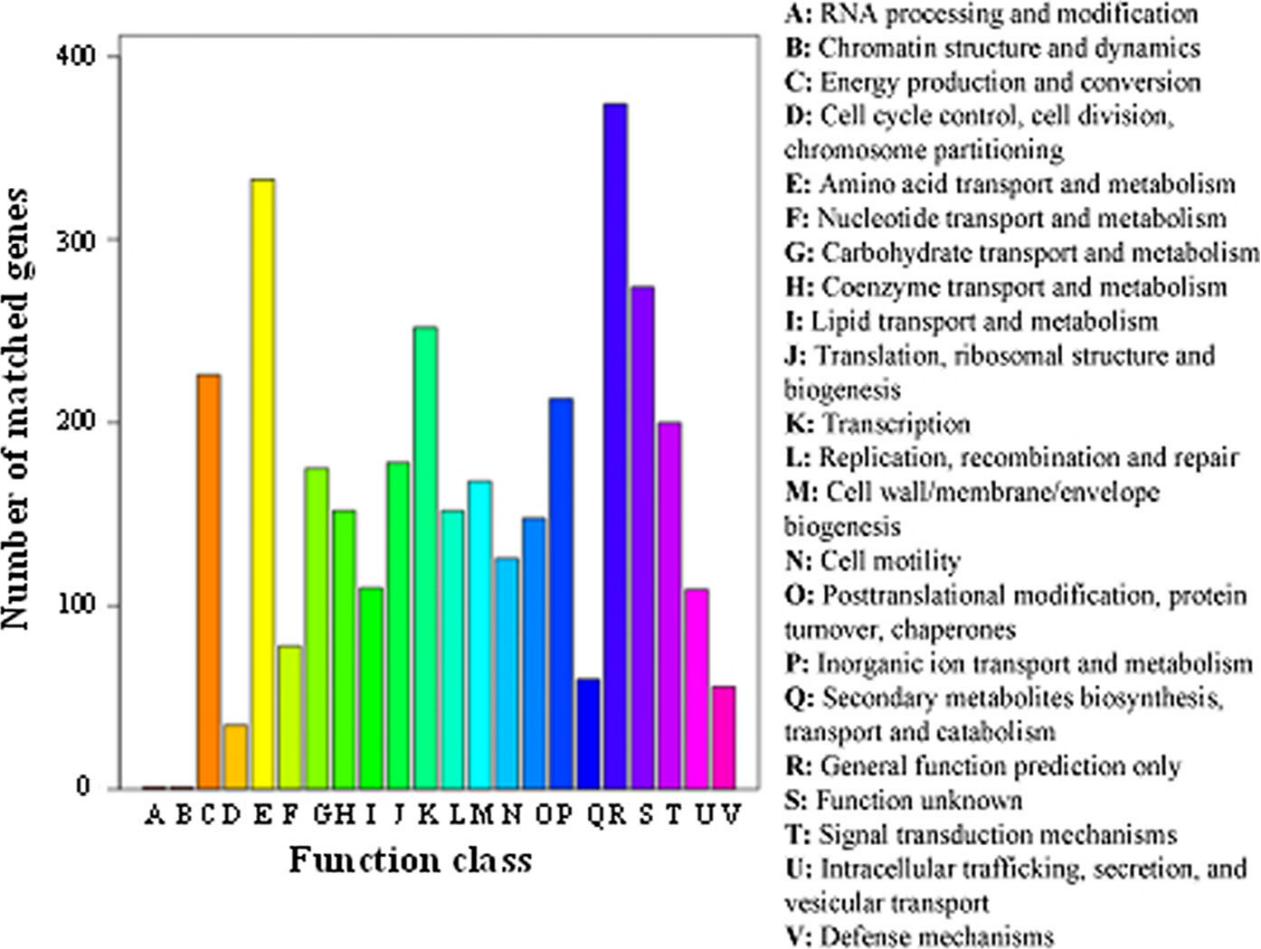
Histogram presentation of clusters of orthologous groups (COGs) classification of all-DEGs

We also assigned the function of all the DEGs by COG analysis. In the COG functional classification, about 65.3 % of the DEGs could be annotated to 3418 functions involved in 22 COG categories, while no DEG was annotated to “extracellular structures”, “nuclear structure”, or “cytoskeleton” (Fig. 4). Among the 22 COG categories, the “General function prediction only” cluster represented the largest group (374 genes), followed by the “Amino acid transport and metabolism”, “Function unknown” and “Transcription” clusters. The “RNA processing and modification”, “Chromatin structure and dynamics” and “Cell cycle control, cell division and chromosome partitioning” represented the smallest clusters predicted by COG.

### KEGG pathway annotation

To identify the biological pathways that are active in bacterial adherence, we mapped the DEGs to reference canonical pathways in KEGG.

Using KEGG, DEGs were assigned to 164 KEGG pathways. Those pathways with the greatest representation by DEGs were “ABC transportersystem”, “Two-component system”, “Glyoxylate and dicarboxylate metabolism” and “Flagellar assembly”. These annotations provide a substantial resource for investigating specific processes, functions and pathways during bacteria adherence.

Among the 164 KEGG pathways, some pathways are known to be closely related to adhesion, for example: “Two-component system”, “Bacterial chemotaxis” and “Flagellar assembly”. Interestingly, the levels of “Bacterial chemotaxis” genes among the different stressed groups was low pH treated group >Cu^2+^ treated group >Pb^2+^ treated group >Hg^2+^ treated group, which was consistent with the extent that adhesion was decreased by these conditions.

Other pathways that displayed expression levels consistent with the extent that adhesion decreased in the stressed groups were also observed, although they have not apparently been reported to be associated with the process of adhesion, such as “Metabolic pathways”, “Microbial metabolism in diverse environments”, “Biosynthesis of secondary metabolites”, and “ABC transporter system”.

## Discussion

Transcriptome sequencing using next-generation technologies provides resources for gene expression profiling studies as well as simultaneous identification of mutations, sequence aberrations, alternative splice variants and RNA editing events (Morozova et al. 2009). The present study focused on the application of next-generation sequencing to transcriptome analysis of *V. alginolyticus* and further understanding of the mechanisms affecting bacterial adhesion. One of the most important aspects in transcriptome analysis is to associate individual sequences and related expression information with biological functions. These annotations provide a resource for further functional characterization of genes during the adhesion of *V. alginolyticus*, and further understanding of how the adhesion process may be influenced by environmental factors.

In the present study, we obtained adhesion affected models through culturing *V. alginolyticus* under stress conditions including Cu^2+^, Pb^2+^, Hg^2+^ and low pH. According to our results, a significant decline in adherence was observed in all stressed groups, while the low pH treated group displayed the lowest adhesion. Interestingly, the results of DEGs analysis showed that low pH treated group yielded the most DEGs, followed by Cu^2+^ treated group, Pb^2+^ treated group and Hg^2+^ treated group, which was consistent with the adhesion phenotype of these groups. These results suggest that these DEGs may be related to bacterial adhesion. Furthermore, the 213 common regulated genes might play important roles in bacterial adhesion, dissecting which may add to understanding of the mechanisms underlying the regulation of adhesion in *V. alginolyticus.* In addition, these 213 common regulated genes were sensitive to the environmental factors tested, and so further analysis of these may reveal how the adhesion process is influenced by environmental factors. Notably, only one up-regulated gene was found in all four treated groups, which encodes the type III secretion protein SctT. It is well known that type III secretion systems delivers many structurally diverse bacterial virulence proteins into plant and animal cells to modulate host cellular functions. How the type III secretion system may participate in influencing the adhesion of *V. alginolyticus* has not yet been reported. Therefore, further research is still necessary.

GO analysis indicated that many of the DEGs were related to biological processes correlated with bacterial adhesion and environmental stress responses, such as “cell motility”, “response to chemical stimulus” and “establishment of localization”. KEGG analysis suggested that many of the DEGs were concentrated in biological regulation, cellular process, localization, establishment of localization and metabolic processes, which meant the stress treatments could affect intricate biological process (including adhesion) of *V. alginolyticus.*

Among the 164 KEGG pathways, some pathways may be closely related to the adhesion process. For example, “Two-component system”, “Bacterial chemotaxis” and “Flagellar assembly” can be closely related to the motility and adherence of bacteria (Mello et al. 2007; Philippe et al. 2000; Victor et al. 2004; Takekawa et al. 2014; Wang et al. 2000; Meadows et al. 1971; DeBoer et al. 1975; Kawagishi et al. 1995; Bordas et al. 1996, 1998; Belas et al. 1982). The process of adhesion of bacteria is connected to the movement of bacteria in response to a chemical stimulus. Chemical gradients are sensed through multiple transmembrane receptors, called methyl-accepting chemotaxis proteins (MCPs), which vary in the molecules that they detect. These receptors may bind attractants or repellents directly or indirectly through interaction with proteins of the periplasmic space. The signals from these receptors are transmitted across the plasma membrane into the cytosol, where the two-component system is activated. The two-component system then induces tumbling by interacting with the flagellar switch protein FliM, inducing a change from counter-clockwise to clockwise rotation of the flagellum. Change in the rotation state of a single flagellum can disrupt the entire flagella bundle and cause a tumble. Interestingly, the levels of “Bacterial chemotaxis” genes among different stressed groups was ranked low pH treated group >Cu^2+^ treated group >Pb^2+^ treated group >Hg^2+^ treated group, which was consistent with the extent their adhesion decreased. These results reinforce the hypothesis that the DEGs found in the present study might play important roles in bacterial adhesion.

To our knowledge, this is the first report of the application of next generation sequencing technology to provide an annotated overview of *V. alginolyticus* gene expression and identification of adhesion related DEGs. Taken as a whole, the present study demonstrates that chemical stress of *V. alginolyticus* induces changes in gene expression profiles. This is, to our knowledge, the first analysis of differentially expressed genes in *V. alginolyticus* after chemical stress. Our results demonstrated that some of the changed genes correlate with changes in bacterial adhesion, which validated previous results and offered new clues for further understanding of the mechanism(s) underlying the regulation of adhesion by *V. alginolyticus* and how this may be influenced by environmental factors. However, further research is now needed to further define the significance of these findings.

## Electronic supplementary material

Below is the link to the electronic supplementary material.

Supplementary material 1 (TIFF 44 kb). The adhesion ability to mucus of wild and stressed (including Cu^2+^, Pb^2+^, and Hg^2+^ at different concentrations) *V. alginolyticus*. Data are presented as mean ± S.D. (n = 3). Means of treatments not sharing a common letter are significantly different at *P* < 0.05 as assessed using one-way ANOVA followed by the Dunnett’s test

Supplementary material 2 (PDF 14 kb)

Supplementary material 3 (DOCX 28 kb)
