# Supplementary figures and images for: Investigation of possible molecular mechanisms underlying the regulation of adhesion in Vibrio alginolyticus with comparative transcriptome analysis
Source: Antonie Van Leeuwenhoek. 2015 Mar 1;107(5):1197–206. doi: 10.1007/s10482-015-0411-9 (PMC4387256; doi:10.1007/s10482-015-0411-9)

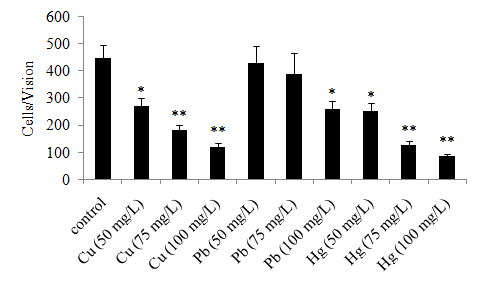

Supplement: Supplementary file 1 — Supplementary material 1 (TIFF 44 kb). The adhesion ability to mucus of wild and stressed (including Cu2+, Pb2+, and Hg2+ at different concentrations) V. alginolyticus. Data are presented as mean ± S.D. (n = 3). Means of treatments not sharing a common letter are significantly different at P < 0.05 as assessed using one-way ANOVA followed by the Dunnett’s test [file 10482_2015_411_MOESM1_ESM.tif]
