# Supplementary material for: Investigation of possible molecular mechanisms underlying the regulation of adhesion in Vibrio alginolyticus with comparative transcriptome analysis
Source: Antonie Van Leeuwenhoek. 2015 Mar 1;107(5):1197–206. doi: 10.1007/s10482-015-0411-9 (PMC4387256; doi:10.1007/s10482-015-0411-9)
Supplement: Supplementary file 2 — Supplementary material 2 (PDF 14 kb) [file 10482_2015_411_MOESM2_ESM.pdf]

Table S1. Primers for QPCR

Gene	Primers for QPCR
aotJ	5'-ATACGCACTGATTCCAAACAAA-3' (F)
	5'-CAATGACGGACCAACAAACTC-3' (R)
aotM	5'-CAGAAGTATTGGAGTTTGGCGTG-3' (F)
	5'-CAGCGATGCGATAACCCTTG-3' (R)
livM	5'-CAGAAGTATTGGAGTTTGGCGTG -3' (F)
	5'-CAGCGATGCGATAACCCTTG-3' (R)
oppC	5'-TTTTGGCAACAACATCTACCTGA -3' (F)
	5'-GTTGAACACCAAGCCCTAAGAA-3' (R)
oppF	5'-CGCAACTTAAACGGGCAGAG -3' (F)
	5'-CGCACTAACGGGTTCATCACA-3' (R)
proW	5'-CACAAGGTGTAGGTGCTGACG -3' (F)
	5'-TGTTCCACCAACCAGTTCAATC-3' (R)
tupA	5'-CGAACCGAACTTTGGTGGCT-3' (F)
	5'-TTGTTGGGTAACGCTCAGGAT-3' (R)
tupB	5'-AACCCGCTTTCCTCTACTCGC-3' (F)
	5'-TGCTTGATAAGAAGAAGTTTAGT-3' (R)

Table S2. Gene number with various coverage percentages

Coverage percentage	control (Number)	Cu (Number)	Pb (Number)	Hg (Number)	low pH (Number)
90%-100%	2233	1959	1911	2107	1945
80%-90%	605	630	591	631	663
70%-80%	393	380	386	337	450
60%-70%	244	262	307	254	326
50%-60%	174	211	176	197	214
40%-50%	114	156	173	163	135
30%-40%	82	153	151	107	106
20%-30%	72	110	114	98	78
10%-20%	62	80	91	67	57
0%-10%	15	21	24	17	13
