# Supplementary material for: Investigation of possible molecular mechanisms underlying the regulation of adhesion in Vibrio alginolyticus with comparative transcriptome analysis
Source: Antonie Van Leeuwenhoek. 2015 Mar 1;107(5):1197–206. doi: 10.1007/s10482-015-0411-9 (PMC4387256; doi:10.1007/s10482-015-0411-9)
Supplement: Supplementary file 3 — Supplementary material 3 (DOCX 28 kb) [file 10482_2015_411_MOESM3_ESM.docx]

**Table S3. List of common regulated genes**

| **gene** | **Cu** | **Pb** | **Hg** | **low pH** | **gene** | **Cu** | **Pb** | **Hg** | **low pH** | **gene** | **Cu** | **Pb** | **Hg** | **low pH** |
| --- | --- | --- | --- | --- | --- | --- | --- | --- | --- | --- | --- | --- | --- | --- |
| K11688 | -3.99 | -3.56 | -3.16 | -2.95 | K00648 | -1.69 | -2.79 | -1.35 | -1.54 | K00140 | -4.99 | -7.50 | -6.20 | -4.18 |
| K10125 | -1.74 | -3.06 | -1.59 | -1.12 | K00680 | -3.04 | -1.69 | -2.04 | -2.24 | K00626 | -4.98 | -8.45 | -7.42 | -4.71 |
| K13590 | -2.69 | -2.14 | -1.42 | -1.50 | K04751 | -1.32 | -1.41 | -1.05 | -1.37 | K13638 | -3.65 | -3.98 | -3.72 | -2.38 |
| K03406 | -1.58 | -1.25 | -1.19 | -3.42 | K08307 | -1.64 | -2.36 | -2.13 | -2.11 | K00253 | -3.00 | -3.56 | -3.88 | -1.13 |
| K10025 | -3.17 | -3.37 | -2.54 | -3.44 | K03299 | -1.78 | -1.79 | -1.07 | -1.05 | K01969 | -4.85 | -3.59 | -6.02 | -2.63 |
| K10022 | -2.08 | -1.76 | -1.16 | -3.60 | K07275 | -3.24 | -3.88 | -3.77 | -3.53 | K01998 | -3.50 | -13.00 | -4.93 | -1.48 |
| K10024 | -2.52 | -2.79 | -2.09 | -2.36 | C408_0812 | -2.40 | -1.15 | -1.10 | -2.92 | K01997 | -3.11 | -13.28 | -4.96 | -1.57 |
| K10023 | -1.96 | -1.87 | -2.10 | -1.20 | K03442 | -1.18 | -1.57 | -1.85 | -1.75 | K01897 | -5.07 | -6.95 | -5.92 | -2.99 |
| K03466 | -1.53 | -1.35 | -1.06 | -1.15 | C408_0890 | -4.92 | -4.59 | -3.90 | -3.90 | K01995 | -3.17 | -14.19 | -6.72 | -5.78 |
| C408_0191 | -1.96 | -1.87 | -3.10 | -2.20 | K01607 | -4.13 | -2.33 | -1.94 | -3.46 | K00311 | -5.17 | -8.55 | -7.02 | -5.13 |
| C408_0192 | -4.35 | -4.82 | -4.20 | -5.04 | K05879 | -2.15 | -1.15 | -1.28 | -4.38 | K03522 | -3.73 | -6.22 | -4.42 | -3.61 |
| K09681 | -2.24 | -2.75 | -2.16 | -1.19 | K00820 | -2.06 | -1.03 | -1.14 | -2.36 | K03585 | -3.14 | -4.11 | -3.77 | -3.10 |
| K03406 | -3.99 | -4.09 | -2.89 | -2.29 | K02468 | -3.74 | -1.05 | -1.35 | -1.43 | K03296 | -2.90 | -1.82 | -1.83 | -1.02 |
| K03704 | -3.46 | -4.21 | -2.62 | -3.24 | K14445 | -2.96 | -1.22 | -1.06 | -1.85 | K00837 | -4.62 | -3.64 | -2.89 | -2.69 |
| K12573 | -3.99 | -3.51 | -2.39 | -1.77 | K00957 | -3.43 | -1.02 | -1.27 | -2.65 | K11920 | -1.25 | -3.79 | -3.40 | -2.59 |
| K01724 | -2.35 | -3.23 | -1.94 | -3.52 | K02303 | -4.10 | -2.07 | -2.13 | -2.78 | K16077 | -2.97 | -1.69 | -1.15 | -1.66 |
| K00500 | -4.68 | -5.38 | -3.90 | -4.31 | K03406 | -2.25 | -1.56 | -1.39 | -1.21 | K03309 | -3.07 | -2.12 | -1.73 | -1.09 |
| K01907 | -4.15 | -3.33 | -3.17 | -2.31 | K02022 | -1.53 | -1.49 | -1.13 | -1.19 | K02406 | -4.71 | -3.70 | -3.79 | -1.08 |
| K03406 | -4.28 | -3.64 | -1.58 | -1.24 | C408_1052 | -2.58 | -2.37 | -1.66 | -3.99 | K02395 | -2.30 | -1.87 | -1.98 | -1.03 |
| C408_0350 | -1.60 | -1.33 | -1.03 | -1.33 | K00020 | -1.42 | -1.52 | -1.13 | -1.68 | K02393 | -2.85 | -2.59 | -2.09 | -1.33 |
| K03286 | -4.00 | -2.96 | -2.95 | -1.16 | K00903 | -2.67 | -2.38 | -1.82 | -1.94 | K03415 | -2.61 | -2.15 | -2.08 | -1.04 |
| K02275 | -1.79 | -1.67 | -1.22 | -1.87 | C408_1079 | -3.30 | -4.76 | -2.56 | -1.28 | K11717 | -3.59 | -3.68 | -2.73 | -3.62 |
| K01826 | -1.59 | -1.91 | -1.28 | -1.29 | K03606 | -4.59 | -3.15 | -3.85 | -1.96 | K00257 | -2.95 | -2.05 | -1.50 | -3.05 |
| **gene** | **Cu** | **Pb** | **Hg** | **low pH** | **gene** | **Cu** | **Pb** | **Hg** | **low pH** | **gene** | **Cu** | **Pb** | **Hg** | **low pH** |
| K08154 | -2.70 | -3.09 | -1.86 | -1.74 | C408_1098 | -1.46 | -1.48 | -1.34 | -1.29 | K03577r | -3.52 | -1.86 | -1.82 | -3.54 |
| K03406 | -5.81 | -4.82 | -4.60 | -1.19 | C408_1135 | -2.60 | -1.14 | -1.13 | -1.33 | K01246 | -1.87 | -1.75 | -4.18 | -2.92 |
| C408_0389 | -2.88 | -2.14 | -3.18 | -4.12 | K02000 | -3.64 | -2.04 | -1.71 | -1.78 | K03970 | -2.59 | -1.60 | -1.58 | -3.07 |
| K01952 | -1.22 | -1.48 | -1.71 | -1.69 | K02001 | -3.27 | -2.25 | -1.33 | -1.94 | K02000 | -4.18 | -2.10 | -2.37 | -2.85 |
| K03309 | -3.80 | -2.19 | -2.40 | -1.66 | K02002 | -2.78 | -2.66 | -1.42 | -2.50 | K02001 | -2.22 | -1.97 | -1.77 | -2.64 |
| K01740 | -2.23 | -1.30 | -1.08 | -4.06 | K00108 | -3.95 | -2.82 | -2.05 | -2.84 | K03310 | -5.07 | -4.79 | -4.88 | -1.22 |
| K13636 | -3.62 | -2.24 | -1.33 | -1.27 | K00130 | -4.35 | -2.52 | -2.37 | -3.01 | K00633 | -2.40 | -1.75 | -1.34 | -1.06 |
| K00285 | -1.89 | -2.87 | -1.88 | -1.95 | K02167 | -5.03 | -3.23 | -2.62 | -2.81 | C408_1699 | -1.98 | -1.48 | -1.28 | -2.60 |
| K03310 | -2.36 | -2.61 | -2.09 | -1.77 | K13590 | -1.80 | -1.99 | -1.27 | -1.80 | K01800 | -3.62 | -3.99 | -4.87 | -3.63 |
| K03601 | -1.05 | -1.69 | -1.42 | -1.51 | K00059 | -3.43 | -6.12 | -5.09 | -3.34 | K00451 | -4.65 | -4.93 | -4.05 | -4.02 |
| K01637 | -2.98 | -3.92 | -2.29 | -1.95 | K00020 | -3.23 | -4.22 | -4.19 | -4.84 | K02035 | -5.56 | -6.50 | -5.96 | -6.12 |
| K01638 | -3.51 | -3.06 | -2.42 | -2.58 | K01692 | -4.66 | -6.05 | -5.60 | -3.86 | K02033 | -4.44 | -4.32 | -5.29 | -3.35 |
| C408_0642 | -4.90 | -2.68 | -3.31 | -1.51 | K00257 | -4.00 | -5.64 | -5.93 | -2.27 | K02034 | -2.91 | -2.47 | -2.18 | -3.24 |
| K02031 | -3.15 | -4.26 | -3.49 | -3.95 | K01681 | -3.03 | -3.80 | -2.94 | -3.31 | K00219 | -2.16 | -1.19 | -1.18 | -1.44 |
| K02032 | -3.56 | -4.95 | -3.99 | -5.53 | K01659 | -3.78 | -4.38 | -3.91 | -3.62 | K03286 | -3.68 | -3.53 | -3.17 | -1.43 |
| K01262 | -3.24 | -3.51 | -3.09 | -2.30 | K03417 | -3.92 | -3.47 | -2.82 | -4.88 | K00243 | -2.27 | -1.90 | -1.19 | -1.08 |
| K01387 | -4.85 | -3.17 | -3.08 | -1.75 | K05799 | -4.36 | -3.99 | -3.93 | -4.44 | K05832 | -3.04 | -1.83 | -1.67 | -1.73 |
| K02052 | -4.36 | -4.24 | -1.84 | -2.60 | K03228 | 1.29 | 1.35 | 2.41 | 3.42 | K04752 | -3.23 | -3.57 | -1.54 | -2.56 |
| K01714 | -3.79 | -4.17 | -4.38 | -2.93 | K01462 | -2.07 | -3.23 | -2.84 | -1.91 | K03320 | -2.31 | -2.16 | -1.44 | -2.06 |
| K01750 | -4.44 | -4.21 | -3.74 | -3.05 | C408_2690 | -2.38 | -1.82 | -1.51 | -2.29 | K03406 | -1.74 | -1.39 | -1.41 | -1.22 |
| K02055 | -4.54 | -4.11 | -3.65 | -1.81 | K03415 | -3.45 | -3.18 | -2.96 | -1.15 | K07023 | -1.61 | -1.21 | -1.11 | -1.09 |
| K00285 | -4.09 | -4.59 | -4.63 | -1.99 | K00122 | -1.27 | -1.44 | -1.12 | -1.47 | K07552 | -1.26 | -1.49 | -1.32 | -2.11 |
| K12658 | -4.55 | -5.35 | -4.10 | -3.70 | K06076 | -2.36 | -2.06 | -1.85 | -3.21 | K06199 | -5.39 | -2.46 | -4.02 | -1.60 |
| K14519 | -3.57 | -4.36 | -3.38 | -3.25 | K07053 | -3.07 | -2.93 | -2.57 | -3.22 | K00540 | -2.40 | -2.44 | -1.98 | -3.62 |
| K14062 | -9.39 | -4.75 | -3.26 | -6.50 | K10253 | -2.69 | -1.52 | -2.00 | -1.56 | C408_3887 | -2.01 | -1.94 | -1.12 | -3.13 |
| **gene** | **Cu** | **Pb** | **Hg** | **low pH** | **gene** | **Cu** | **Pb** | **Hg** | **low pH** | **gene** | **Cu** | **Pb** | **Hg** | **low pH** |
| K01667 | -2.87 | -2.03 | -1.25 | -3.79 | K15578 | -1.86 | -12.29 | -2.07 | -3.36 | K04774 | -1.51 | -1.02 | -1.04 | -1.89 |
| K01638 | -3.18 | -2.38 | -2.93 | -1.54 | K00362 | -2.17 | -2.10 | -1.55 | -1.75 | K03558 | -1.33 | -1.25 | -1.18 | -1.86 |
| C408_1806 | -3.09 | -1.97 | -1.57 | -4.59 | K01113 | -3.10 | -2.63 | -1.99 | -1.27 | K02688 | -2.98 | -2.06 | -2.03 | -1.38 |
| K01770 | -1.23 | -1.07 | -1.20 | -1.44 | K03406 | -2.52 | -1.34 | -1.21 | -1.79 | K00128 | -4.20 | -6.67 | -4.47 | -2.46 |
| K01807 | -1.71 | -1.52 | -1.95 | -1.01 | K01409 | -1.58 | -1.20 | -1.70 | -1.01 | K13924 | -1.72 | -1.73 | -1.05 | -1.11 |
| K04069 | -1.21 | -3.06 | -1.99 | -2.08 | K00655 | -1.64 | -3.63 | -1.31 | -1.86 | C408_4101 | -2.89 | -3.60 | -1.51 | -1.45 |
| K01246 | -1.39 | -1.66 | -1.28 | -1.44 | K03406 | -1.71 | -1.48 | -1.24 | -1.87 | K14393 | -4.87 | -5.24 | -4.38 | -4.38 |
| C408_2111 | -1.01 | -1.13 | -1.35 | -1.35 | K15034 | -4.02 | -4.30 | -3.82 | -3.21 | K06447 | -1.96 | -1.75 | -1.11 | -2.42 |
| C408_2112 | -6.31 | -2.73 | -2.07 | -2.13 | K03406 | -3.40 | -2.36 | -2.20 | -1.85 | K00821 | -2.09 | -1.97 | -1.65 | -2.90 |
| K03451 | -5.63 | -4.97 | -5.24 | -3.86 | K00127 | -3.06 | -1.78 | -2.11 | -1.40 | C408_4212 | -1.90 | -1.34 | -1.09 | -1.19 |
| K01782 | -1.96 | -1.54 | -1.07 | -2.95 | K15831 | -2.57 | -1.06 | -2.32 | -1.04 | K03543 | -4.11 | -4.36 | -3.93 | -3.92 |
| K00632 | -2.07 | -1.55 | -1.35 | -2.69 | K02379 | -1.99 | -1.45 | -2.31 | -2.12 | K06075 | -4.21 | -4.80 | -4.01 | -5.21 |
| K02422 | -3.20 | -2.56 | -2.28 | -1.34 | K01552 | -1.40 | -1.61 | -2.36 | -1.83 | K06073 | -1.53 | -2.22 | -2.99 | -1.94 |
| K02407 | -3.07 | -2.80 | -2.55 | -1.10 | K02481 | -2.44 | -1.72 | -1.89 | -1.17 | K03557 | -1.23 | -1.41 | -1.48 | -1.87 |
| K02406 | -2.26 | -1.63 | -1.49 | -1.21 | K05773 | -2.53 | -2.64 | -2.49 | -1.40 | K01895 | -2.48 | -1.91 | -1.19 | -3.82 |
| K02406 | -5.11 | -3.79 | -4.09 | -1.03 | K03704 | -3.55 | -3.32 | -2.50 | -2.17 | C408_4338 | -2.03 | -2.51 | -2.02 | -1.13 |
| K02406 | -5.13 | -3.94 | -4.16 | -1.52 | K06911 | -1.21 | -2.20 | -1.02 | -1.30 | K00390 | -3.44 | -1.05 | -1.05 | -1.49 |
| K02480 | -1.72 | -1.70 | -1.07 | -1.32 | K01175 | -2.55 | -2.69 | -2.13 | -2.35 | K07025 | -2.28 | -4.16 | -1.67 | -2.03 |
| K04015 | -3.33 | -1.96 | -1.18 | -1.03 | K10918 | -2.22 | -1.52 | -2.07 | -2.62 | K01175 | -2.94 | -1.67 | -1.51 | -1.88 |
| K01175 | -4.04 | -3.43 | -2.75 | -2.75 | K00411 | -3.74 | -3.00 | -2.45 | -1.45 | K06889 | -4.20 | -3.67 | -1.64 | -3.12 |
| K03458 | -1.49 | -1.02 | -1.81 | -1.36 | K00407 | -2.12 | -2.42 | -1.78 | -1.02 | C408_4456 | -2.31 | -5.44 | -3.24 | -1.08 |
| C408_2589 | -1.62 | -2.65 | -1.62 | -2.40 | K00405 | -1.50 | -1.96 | -1.50 | -1.07 | K01908 | -2.84 | -2.21 | -1.74 | -1.51 |
| K00680 | -2.83 | -3.07 | -1.60 | -1.33 | K00404 | -2.63 | -2.48 | -2.31 | -1.13 | K01716 | -1.64 | -2.22 | -2.06 | -2.38 |
